# Identification of clinical and virological correlates associated with influenza A candidate vaccine virus (CVV) attenuation in a ferret model

**DOI:** 10.1128/jvi.01023-25

**Published:** 2025-09-17

**Authors:** Claudia Pappas, Nicole Brock, Jessica A. Belser, Troy J. Kieran, Joanna A. Pulit-Penaloza, Xiangjie Sun, Hui Zeng, Li Wang, Bin Zhou, Terrence M. Tumpey, Taronna R. Maines

**Affiliations:** 1 Influenza Division, Centers for Disease Control and Prevention 1242, Atlanta, Georgia, USA; St Jude Children's Research Hospital, Memphis, Tennessee, USA

**Keywords:** zoonotic influenza virus, ferrets, candidate vaccine virus

## Abstract

Influenza A viruses continuously circulate among avian and swine species, posing a persistent threat to public health. The development of influenza candidate vaccine viruses (CVVs) plays a pivotal role in the global strategy for influenza pandemic preparedness. Safety-testing of CVVs for attenuation in ferrets represents a critical step that takes place prior to making these viruses available to vaccine manufacturers. Development of pathogenicity standards is needed to establish acceptable thresholds of disease so that CVV safety can be assessed without the need for comparison to the parental virus. To assess the capacity of diverse CVVs to cause pathogenesis in mammalian hosts, clinical and virological parameters were compiled from CVV assessments in ferrets conducted using consistent methods over approximately 20 years to identify disease parameters most reflective of attenuation compared to wild-type strains. These analyses revealed an overall reduction in ferret weight loss and fever relative to wild-type controls. Viral titers in nasal washes were reduced with limited spread to tissues beyond the respiratory tract. Regression models further support the significance of clinical signs in distinguishing the virulence of wild-type viruses and CVVs. These findings provide support for the development of standardized parameters for assessing pathogenicity of CVVs and their suitability for manufacturers.

**IMPORTANCE:**

The development and safety testing of candidate vaccine viruses (CVVs) against emerging zoonotic influenza strains prior to sharing with vaccine manufacturers is a critical component of influenza pandemic preparedness. The extensive data set reported here provides critical information that will drastically streamline the safety testing process, thereby enabling more efficient CVV assessments and improving public health in the event of an influenza pandemic.

## INTRODUCTION

Influenza A viruses (IAV) continuously circulate among a diversity of mammalian and avian species, with occasional spillover to humans, posing a persistent threat to human health (1). Risk assessment rubrics, such as the Influenza Risk Assessment Tool (IRAT) developed by the Centers for Disease Control and Prevention (CDC), aid in the identification of zoonotic viruses of public health concern (2, 3) and inform resource allocation for public health countermeasures. Vaccines are widely recognized as the most effective defense against IAV but can be challenging to rapidly deploy in the event of novel virus emergence in a population due to lengthy development and production considerations (4). As such, the development of influenza candidate vaccine viruses (CVVs) plays a pivotal role in the global strategy for influenza pandemic preparedness (5). Once CVVs are meticulously chosen, developed, and characterized (6), they become available to national authorities and vaccine manufacturers for stockpiling, further studies, and clinical trials.

Testing CVV attenuation relative to parental wild-type (WT) strains is a component of development and production practices to reduce risk to humans and animals during vaccine manufacturing. There are several tests to evaluate pathogenicity of CVVs *in vitro* and *in vivo*, including trypsin-independent replication in cell culture, gene sequencing, genetic stability, pathogenicity in chickens, and attenuation in ferrets (7). Ferrets are the preferred mammalian model for assessing attenuated phenotypes compared to WT viruses, as they exhibit many clinical signs of IAV infection similar to those observed in humans and are, therefore, valuable for studying the pathogenicity of zoonotic IAV (8). Due to the ever-increasing diversity of zoonotic IAVs crossing species barriers worldwide, there is substantial phenotypic and genetic variability among WT strains that can affect the relative degree of attenuation observed when safety-testing CVVs (9–11). While a few studies have reported CVV attenuation relative to WT virus in ferrets (12–15), there remains a need to identify and standardize attenuation metrics that are broadly applicable when assessing a diverse range of CVVs, thereby supporting the establishment of an acceptable alternative to direct comparison with the parental WT virus.

Achieving safe development and production of IAV CVVs represents a multi-national effort inclusive of CVV-testing laboratories, vaccine manufacturers, and national regulatory authorities (7, 16). In this context, we present aggregated ferret pathogenicity evaluations of 30 CVVs conducted using consistent methods from one laboratory, with CVV attenuation compared to WT strains of similar subtype/clade produced in the same laboratory or elsewhere. Analyses of clinical (weight loss, fever) and virological parameters (viral detection in nasal washes [NW] and tissues) were performed to identify the metrics most consistently associated with attenuation, independent of strain heterogeneity. Collectively, these findings demonstrate consistent attenuation of CVVs compared to WT viruses and support the development of a standardized protocol with established thresholds for assessing pathogenicity and safety testing of future CVVs.

## RESULTS

### IAV WT and CVVs tested at CDC

CDC has performed IAV CVV testing under a consistent experimental protocol for approximately 20 years, with zoonotic subtypes spanning H1 (including a 2009 H1N1 pandemic virus), H2, H3, H5, H7, H9, and H10, highlighting the diversity of viruses that pose a threat to human health (Tables 1 and 2). As IAV CVV safety-testing typically includes demonstrating attenuation in the ferret model (7), multiple WT viruses of public health concern were characterized in ferrets to serve as reference strains to measure relative attenuation of CVVs in this model. With the exception of the LPAI virus A(H7N7), IBCDC-1, which was a CVV generated by classical reassortment (17), all CVVs shown in Table 1 were generated by reverse genetics (15). CVVs typically consist of six internal genes derived from the A/Puerto Rico/8/1934 A(H1N1) (PR8) virus, combined with the hemagglutinin (HA) and neuraminidase (NA) genes from the WT influenza viral target of interest. In the case of H5 or H7 highly pathogenic avian influenza (HPAI) viruses, the sequences corresponding to the multi-basic amino acids (MBAA) in the HA were removed to prevent HA cleavage by ubiquitous cellular proteases.

**TABLE 1:** Wild-type (WT) and candidate vaccine viruses (CVVs) tested by the U.S. CDC^*l*^

Subtype	Clade/subclade	Virus	WT/CVV abbreviation	Virulence in ferrets			Reference
				% weight loss^*j*^	Temp change (^o^C)^*j*^	Clinical signs^*k*^	
**H1N1**		**A/Puerto Rico/8/1934-RG**	**RG-PR8**	**0.8**	**1.6**	**No**	This study
H1N1	6B	**A/Texas/15/2009** ^*a*^	**WT**	6.4	2.6	Yes	(18)
		A/Texas/05/2009-PR8-IDCDC-RG15^*b*^	RG15	0.7	0.7	No	(13)
H1N1v	1C.2.3	**A/Hunan/42443/2015** ^*a*^	**WT**	13.9	0.7	No	(19)
		A/swine/Jiangsu/J004/2018-PR8-IDCDC-RG68A	RG68A	4.2	0.5	No	This study
H1N1v	1A.3.3.3	**A/Ohio/09/2015** ^*a*^	**WT**	9.6	1.6	Yes	(20)
		A/Wisconsin/03/2021-PR8-IDCDC-RG76A	RG76A	0.0	0.5 (2/3)	No	This study
H2N3	North American	**A/swine/Missouri/2124514/2006**	**WT**	13.7	1.7	Yes	(21)
		A/swine/Missouri/2124514/2006)-PR8-IDCDC-RG27	RG27	5.9	1.3	No	(15)
H3N2v	3.2010.1	**A/Ohio/13/2017**	**WT**	13.9	2	Yes	(22)
		A/Ohio/13/2017-PR8-IDCDC-RG74A^*c*^	RG74A	2	0.7	No	This study
H3N8	Eurasian	**A/chicken/Guangdong-Shantou/481/2022^*l*^**	**WT**	4.3	1.75 (2/3)	Yes	(23)
		A/Henan/4-10CNIC/2022-PR8-IDCDC-RG79A	RG79A	4.2	0.5	No	This study
H5N1	1	**A/Vietnam/1203/2004**	**WT**	15.9	1.4	Yes	(24)
		A/Vietnam/1203/04-PR8-IDCDC-RG	CDC-RG	2.7	0.9	No	This study
H5N1	2.1.3.2	**A/Indonesia/05/2005**	**WT**	16.7	1.4	Yes	(25)
		A/Indonesia/05/2005 PR8-IDCDC-RG2	RG2	5.1	0.7	No	This study
H5N1	2.2.1	**A/Egypt/N03072/2010**	**WT**	9.1	1.8	Yes	(26)
		A/Egypt/N03072/2010(H5N1)-PR8-IDCDC-RG29	RG29	1.2	1.0	No	(15)
H5N1	2.3.2.1a	**A/duck/Vietnam/NCVD-1206/2012** ^*a*^	**WT**	6.5	2.1	No	(26)
		A/Hubei/1/2010(H5N1)-PR8-IDCDC-RG30	RG30	3.5	1.1	No	(15)
		A/Hubei/1/2010(H5N1)-PR8-IDCDC-RG49B^*d*^	RG49B	1.7	1.1	No	This study
		A/Hubei/1/2010(H5N1)-PR8-IDCDC-RG51B^*e*^	RG51B	0.6 (2/3)	0.8	No	This study
		A/duck/Bangladesh/17D1012/2018-PR8-IDCDC-RG63A	RG63A	6.5	0.2	No	This study
H5N1	2.3.2.1c 2.3.2.1f	**A/duck/Vietnam/NVCD-2848/2013** ^*a*^	**WT**	10.0	2.4	No	(26)
		A/chicken/Ghana/20/2015-PR8-IDCDC-RG75A	RG75A	0.2 (1/3)	1.9 (2/3)	No	This study
H5N6	2.3.4.4a	**A/Sichuan/26211/2014**	**WT**	14.2	2.3	Yes	(27)
		A/Sichuan/26211/2014 (H5N6)-PR8-IDCDC-RG42A	RG42A	5.2	1.0	No	This study
H5N1	2.3.4.4b	**A/American Wigeon/SC/22-000345-001/2021**	**WT**	2.2 (2/3)	1.1	No	(28)
H5N8		A/Astrakhan/3212/2020-like IDCDC-RG71A	RG71A	0	0.85	No	This study
H5N6		A/Hunan/10117/2021-PR8-IDCDC-RG77A	RG77A	3.1 (2/3)	0.6	No	This study
H5N1		A/American Wigeon/South Carolina/22-000345-001/2021	RG78A	2.2	0.8	No	This study
H5N1		A/chicken/Ghana/AVL-763_21VIR7050-39/2021-IDCDC-RG80A	RG80A	3.2	0.5	No	This study
H5N8	2.3.4.4c	**A/gyrfalcon/Washington/41088-6/2014**	**WT**	5.3	1.5	No	(29)
		A/gyrfalcon/Washington/41088-6/2014-PR8-IDCDC-RG43B	RG43B	5.5	0.4	No	(15)
H5N2		A/gyrfalcon/Washington/41088-6/2014-PR8-IDCDC-RG47B^*f*^	RG47B	0.8	1.1	No	(15)
H5N6	2.3.4.4	**A/duck/Bangladesh/19D770/2017**	**WT**	13.3	2.2	Yes	(27)
	2.3.4.4g	A/chicken/Vietnam/RAHO4-CD-20-421/2020-PR8-IDCDC-RG69A	RG69A	1.9 (1/3)	0.3	No	This study
H7N7	Eurasian	**A/Netherlands/219/2003**	**WT**	19.8	2.5	Yes	(30)
		A/mallard/Netherlands/12/2000-PR8-IDCDC-1^*g*^	IBCDC-1	4.5	1.8	No	(17)
H7N9	Eurasian	**A/Anhui/1/2013** ^*a*^	**WT**	11	1.5	Yes	(31)
		A/Shanghai/2/2013-PR8-IDCDC-RG32A^*a*^	RG32A	1.3	1.5	No	(32)
H7N9	Yangtze River Delta	**A/Hong Kong/4553/2016**	**WT**	11.6	1.6	Yes	(33)
		A/Hong Kong/125/2017-PR8-IDCDC-RG56B	RG56B	4.7	1.6 (2/3)	No	This study
		**A/Guangdong/17SF003/2016-like PR8-IDCDC-RG56N** ^*h*^	RG56N	2.7	1.2	No	This study
H7N9	Yangtze River Delta	**A/Guangdong/17SF003/2016**	**WT**	18.2	2	Yes	(33)
		A/Gansu/23277/2019-PR8-IDCDC-RG64A	RG64A	2.2	0.5 (2/3)	No	This study
H9N2	G1	**A/Hong Kong/1073/1999**	**WT**	3.7 (1/3)	1.4	Yes	(34)
		A/Oman/2747/2019-PR8-IDCDC-RG66A	RG66A	1.2 (2/3)	0.6	No	This study
H9N2	G9 (B Y280)	**A/Anhui-Lujiang/39/2018**	**WT**	8.5	1.8	Yes	(34)
		A/Anhui-Lujiang/39/2018-PR8-IDCDC-RG61A	RG61A	1.5	1.7	No	This study
H10N7	Eurasian	**A/harbor seal/Germany/PV20762_TS/2014^*i*^**	**WT**	4-18^*i*^	nd^*l*^	Yes	(35)
H10N3		A/Jiangsu/428/2021-PR8-IDCDC-RG73A	RG73A	1.7	2.8 (2/3)	No	This study

^*a*^ Ferrets were intranasally inoculated with 10^6^ PFU of virus, A/Ohio/13/2017 was intranasally inoculated with 10^5^ PFU.

^*b*^ Contains Q223R in HA.

^*c*^ HA has D190N/D225G/P227S modifications.

^*d*^ HA contains the ectodomain from A/Hubei/1/2010, and the remainder from the HA of PR8.

^*e*^ RG30-like with PB1 from H1N1pdm09 and remainder of genes from PR8.

^*f*^ Contains N2 from a representative North American virus.

^*g*^ HA and NA from low pathogenicity virus donors, generated by classic reassortment.

^*h*^ Contains two amino acid changes in the antigenic site that reflect those found in the highly pathogenic avian influenza (HPAI) virus.

^*i*^ Weight loss on days 3 and 7 post inoculation reported by van den Brand JM et al. (35).

^*j*^ Number of ferrets that exhibited weight loss (expressed as mean maximum percentage) or temperature rise (expressed as mean rise above baseline in °C) from preinoculation values/total number of ferrets (in parenthesis); 3/3 unless otherwise specified.

^*k*^ Clinical signs observed days 1–14 p.i., defined by the absence (no) or presence (yes) of one or more of the following symptoms: lethargy score of 1 for at least 2 days, or score of 2 for one or more days (36), respiratory symptoms (sneezing, wheezing, dyspnea), nasal (nasal discharge), diarrhea and neurological (incoordination).

^*l*^ For A/chicken/Guangdong-Shantou/481/2022 and A/harbor seal/Germany/PV20762_TS/2014 viruses, ferrets were inoculated with 10^6^ TCID_50_ intranasal or intratracheally, respectively. Data for A/chicken/Guangdong-Shantou/481/2022 and A/harbor seal/Germany/PV20762_TS/2014 were not obtained from CDC laboratories. nd, not determined. Wild-type viruses that were used as reference are in bold.

**TABLE 2:** Accession numbers for virus sequences

Virus	CVV abbreviation	GenBank accession ID (HA/NA)
A/Puerto Rico/8/1934-RG	RG-PR8	PX102064, PX102065, PX102066, PX102067, PX102068, PX102069, PX102070, PX102071
A/swine/Jiangsu/J004/2018-PR8-IDCDC-RG68A	RG68A	ON856564/ON856565
A/Wisconsin/03/2021-PR8-IDCDC-RG76A	RG76A	OQ871527/OQ871528
A/Ohio/13/2017-PR8-IDCDC-RG74A	RG74A	OQ821661/OQ821662
A/Henan/4-10CNIC/2022-PR8-IDCDC-RG79A	RG79A	PX070140/PX070141
A/Vietnam/1203/04-PR8-IDCDC-RG	CDC-RG	PX093045/PX093046
A/Indonesia/05/2005 PR8-IDCDC-RG2	RG2	PX093047/PX093048
A/Hubei/1/2010(H5N1)-PR8-IDCDC-RG49B	RG49B	PX093049/PX093050
A/Hubei/1/2010(H5N1)-PR8-IDCDC-RG51B	RG51B	CY103897/CY098760
A/duck/Bangladesh/17D1012/2018-PR8-IDCDC-RG63A	RG63A	OP053385/OP053386
A/chicken/Ghana/20/2015-PR8-IDCDC-RG75A	RG75A	OQ830821/OQ830822
A/Sichuan/26211/2014 (H5N6)-PR8-IDCDC-RG42A	RG42A	MG930770/MG930771
A/Astrakhan/3212/2020-like IDCDC-RG71A	RG71A	OM403993/OM403994
A/Hunan/10117/2021-PR8-IDCDC-RG77A	RG77A	OQ944350/OQ944351
A/American Wigeon/South Carolina/22-000345-001/2021	RG78A	OR051630/OR051629
A/chicken/Ghana/AVL-763_21VIR7050-39/2021-IDCDC-RG80A	RG80A	OQ924975/OQ924976
A/chicken/Vietnam/RAHO4-CD-20-421/2020-PR8-IDCDC-RG69A	RG69A	OQ673101/OQ673102
A/Hong Kong/125/2017-PR8-IDCDC-RG56B	RG56B	CY235363/CY235364
A/Guangdong/17SF003/2016-like PR8-IDCDC-RG56N	RG56N	MG930768/MG930769
A/Gansu/23277/2019-PR8-IDCDC-RG64A	RG64A	MW403506/MW403507
A/Oman/2747/2019-PR8-IDCDC-RG66A	RG66A	OQ719942/OQ719943
A/Anhui-Lujiang/39/2018-PR8-IDCDC-RG61A	RG61A	MW403504/MW403505
A/Jiangsu/428/2021-PR8-IDCDC-RG73A	RG73A	PX070138/PX070139

To support high yield in eggs, which is the typical method for vaccine development, additional mutations or modifications, none of which result in gain-of-function in mammalian cells, were introduced into certain CVVs listed in Table 1. Collectively, the use of reverse genetics with a 2:6 WT to PR8 backbone configuration represents a robust, safe, and consistent approach for the rapid generation of CVVs to support pandemic preparedness (7).

### Clinical signs of infection of IAV CVVs in ferrets

**Fig 1:**
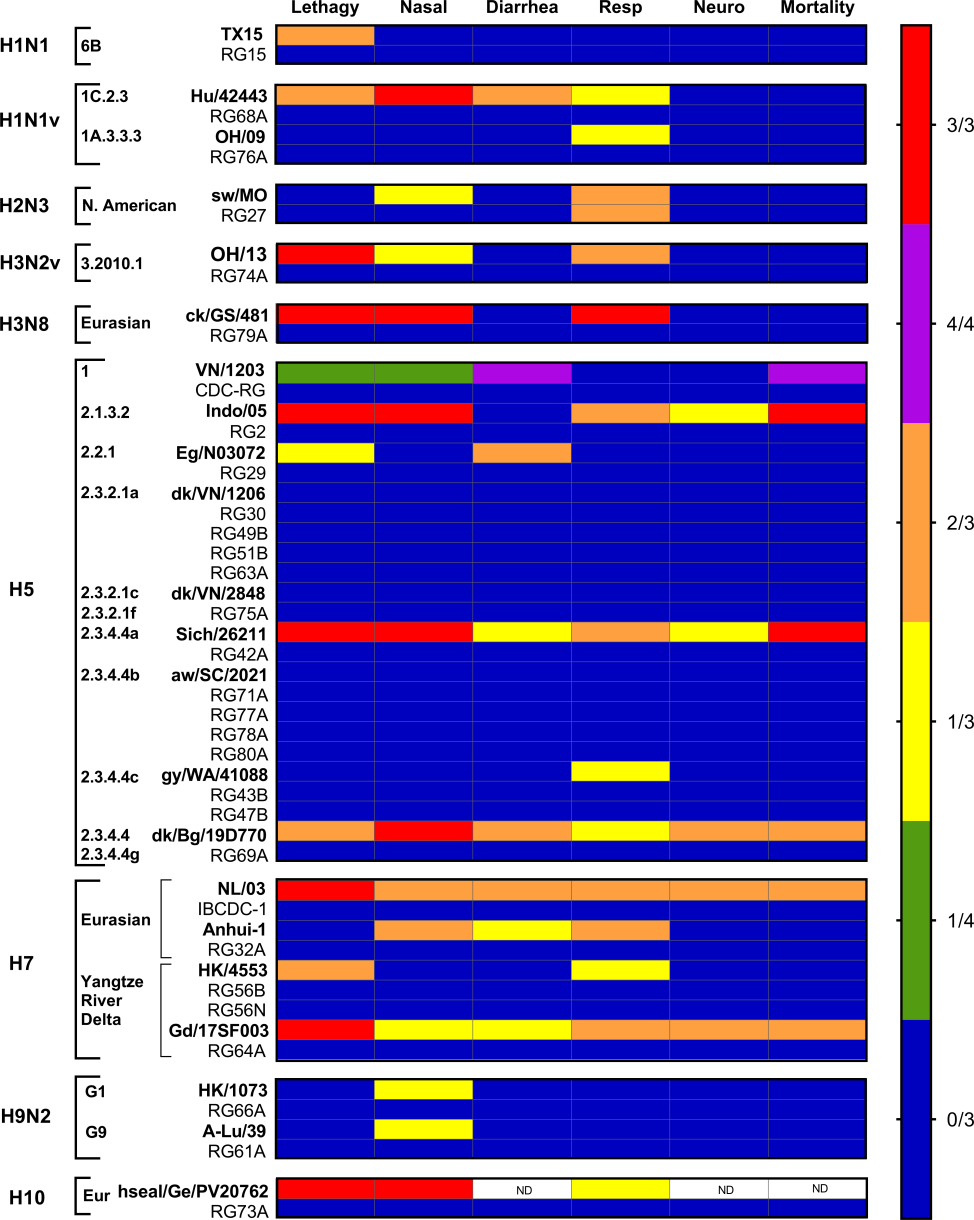
Frequency of clinical signs in ferrets following inoculation with either wild-type or CVV. Viruses are categorized by subtype, clade, and subclade, and the presence of clinical symptoms and number of ferrets affected is indicated by color: blue for absence of symptoms in a total of 3 ferrets (0/3), green for 1 out of 4, yellow for 1 out of 3, orange for 2 out of 3, purple for 4/4, and red for 3 out of 3.

CVVs typically exhibit reduced clinical signs of infection in ferrets relative to parental WT strains, attributed primarily to the presence of a PR8 virus backbone and removal of the MBAA cleavage site in the HA when present (7, 15). All ferrets inoculated with the CVVs listed in Table 1 survived the infection as did RG-PR8-inoculated ferrets, whereas lethal phenotypes were reported among many of the HPAI parental WT strains. Furthermore, the frequency of clinical signs of infection (encompassing lethargy, diarrhea, nasal discharge, respiratory and neurological symptoms, and mortality) was reduced when ferrets were inoculated with CVVs compared with their matched WT viruses (Fig. 1). However, no systematic evaluation has been performed to assess the relative degree of attenuation across a diverse panel of CVVs. To this end, we aggregated data from the viruses shown in Table 1 and compared the relative differences in key clinical parameters between WT and CVV strains, with an aim to identify which recorded parameters during CVV standard pathotyping assessments were most substantially reduced compared to WT.

**Fig 2:**
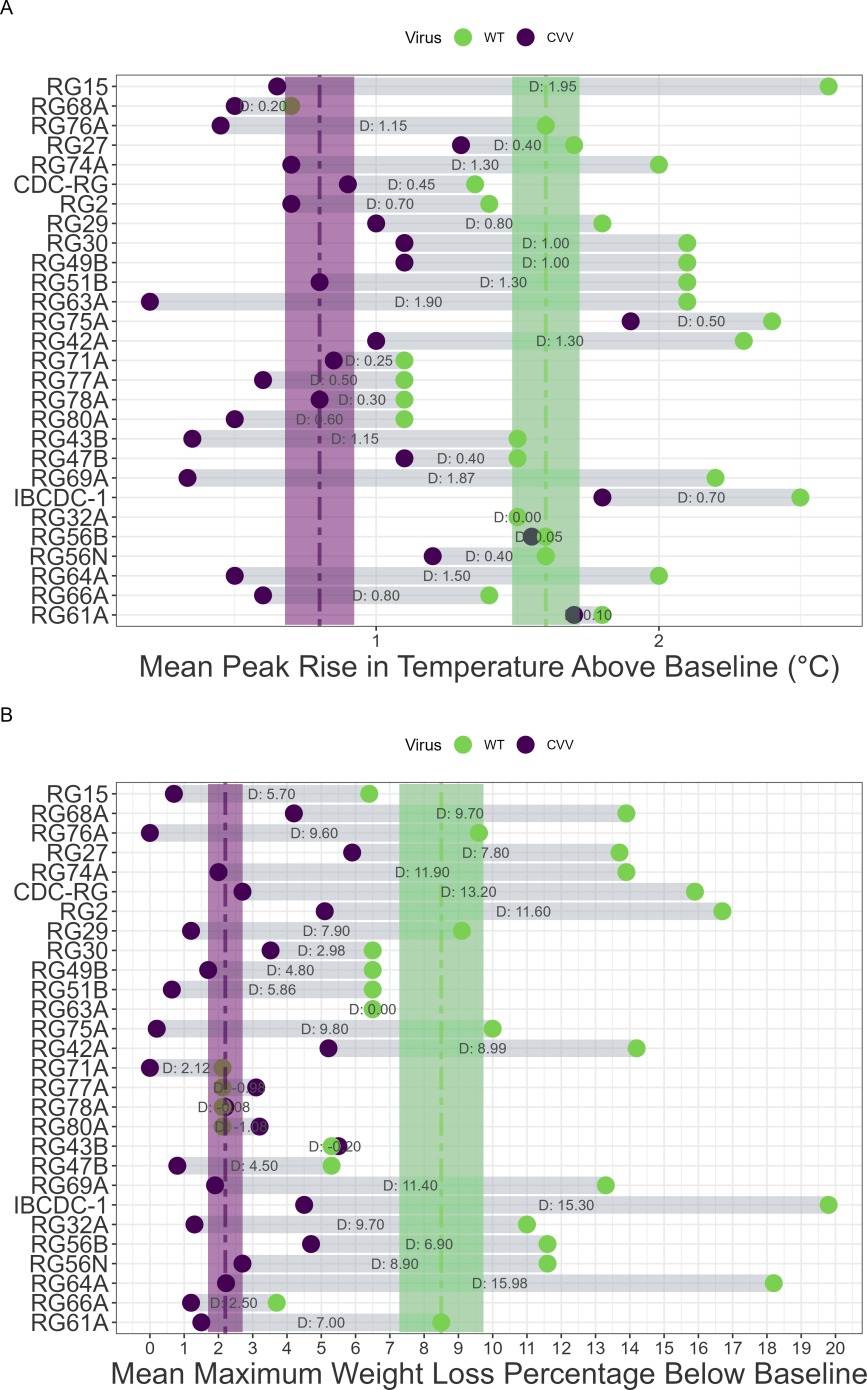
Temperature rise and weight loss comparisons between paired wild-type (WT) and candidate vaccine viruses (CVVs). Mean peak rise in temperature (**A**) and mean maximum weight loss percentage (**B**) are shown as dots connected by the distance between the measurements. The dashed lines represent median values of the per-virus mean values of all CVV or WT viruses tested, while shaded areas (purple [CVV] or green [WT]) indicate standard error. D: indicates the log differences between the measurements. When values were identical between CVV and WT groups, only one dot is shown.

Elevated temperatures in ferrets is a common clinical sign associated with zoonotic IAV infection. All except one of the WT viruses included here had a mean peak temperature of >1°C during days 1–9 post-inoculation (p.i.), with a median rise over baseline across all viruses of 1.7°C (Fig. 2A). CVVs consistently had lower mean peak temperatures than their paired parental WT viruses (median value 0.8°C) with one exception (RG32A), though the degree of reduction varied extensively depending on the parental strain, from 0.1 to 2.0°C. Differences between mean peak rises in temperature were statistically significant between WT and CVV groups (*P* = 4e-8, Table S1), further supporting that CVVs were consistently associated with detectable and meaningful reductions in peak temperature readings during acute infection.

Weight loss is a frequently measured parameter to assess morbidity in laboratory animals (37). While parental strains demonstrated a wide range of weight loss (approaching 20% below baseline), CVVs tested all had mean values < 6%, indicating generally mild morbidity (Fig. 2B). Similar to temperature, the range of attenuated weight loss varied on a per-strain basis. Despite the heterogeneity of maximum weight loss reported among WT strains, mean differences were statistically significant between WT and CVV groups (*P* = 1.3e-7, Table S1). Limiting this analysis to only H5 subtype viruses (inclusive of A(H5N1), A(H5N2), A(H5N6), and A(H5N8) viruses) resulted in comparable results as the entire data set (Table S2), supporting that attenuation of these clinical parameters were maintained across different data sets.

### Virus replication of IAV CVVs in ferrets

**Fig 3:**
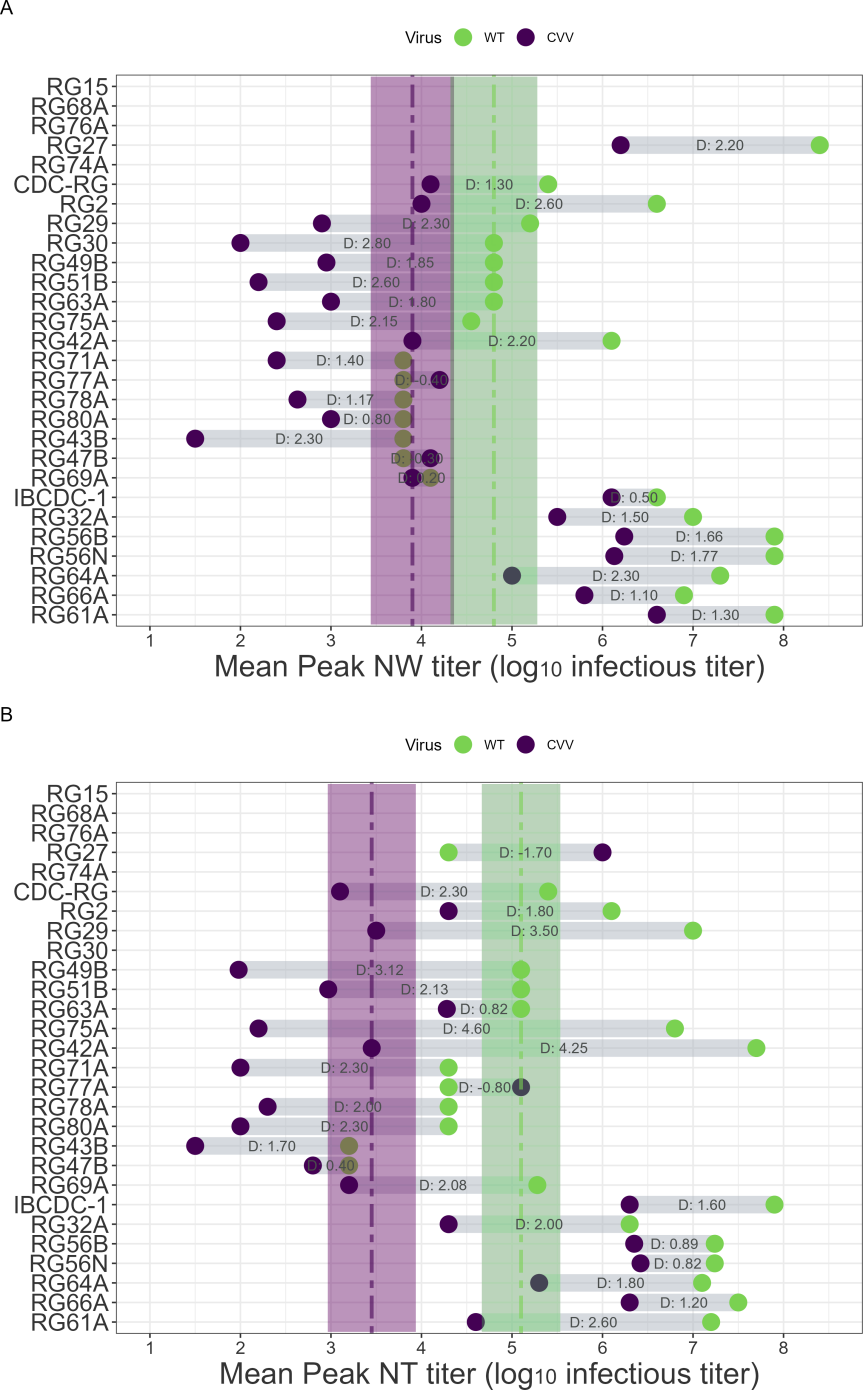
Upper respiratory tract virus titer comparisons between paired wild-type (WT) and candidate vaccine viruses (CVVs). Mean peak virus titers in nasal wash (NW, **A**) and in nasal turbinates (NT, **B**) are shown as dots connected by the distance between the measurements. The dashed lines represent median values of the per-virus mean values of all CVV or WT viruses tested, while shaded areas (purple [CVV] or green [WT]) indicate standard error. D indicates the log differences between the measurements. Missing values indicate a mismatch in titration units between WT and CVV and were excluded from analysis.

The PR8 virus backbone is known for causing mild illness in ferrets although it can replicate moderately in the upper respiratory tract of ferrets up to day 5 p.i., with minimal levels of virus detected in the lungs (12, 38). We first assessed relative differences between viral titers of paired WT and CVVs in two upper respiratory tract specimens: NW (mean peak titers days 1–7 p.i.) and nasal turbinate tissue (mean titers on day 3 p.i.). Attenuation of peak NW viral titer was observed in all but one pair, though the degree of attenuation varied in a strain-specific manner (Fig. 3A). Mean differences in peak NW titer were statistically significant between WT and CVV groups (*P* = 1.5e-3, Table S1; Fig. 3A). Comparison of nasal turbinate viral titers revealed a similar pattern (Fig. 3B), with greater distance between both mean and median titers of groups, with statistical significance of *P* = 2.7e-4 (Table S1). Statistical significance between differences for both mean peak NW and mean day 3 nasal turbinate titers was maintained when aggregated viruses were limited to the H5 subtype (Table S2).

**Fig 4:**
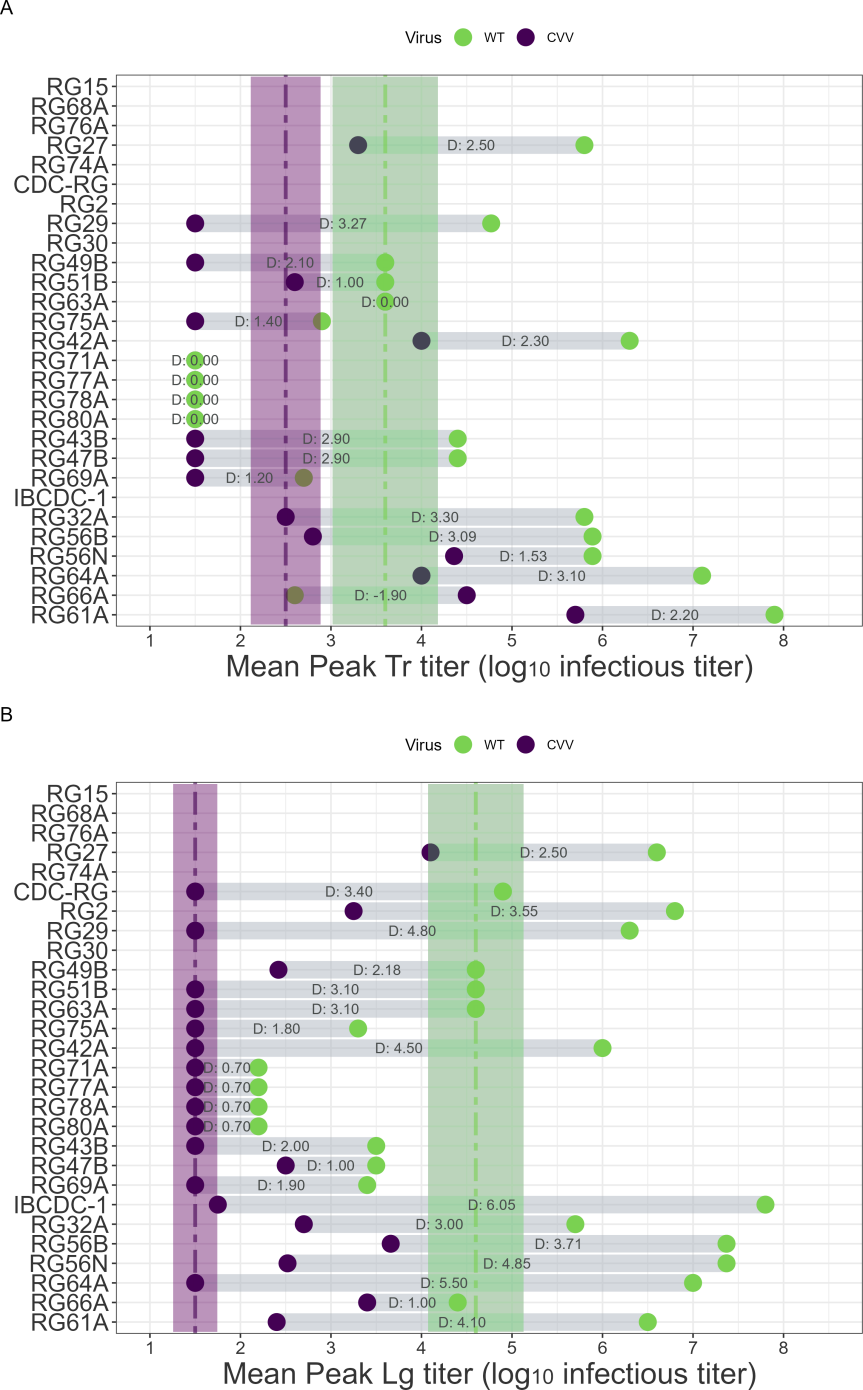
Lower respiratory tract virus titer comparisons between paired wild-type (WT) and candidate vaccine viruses (CVVs). Mean peak titers in trachea (Tr, **A**) and in lung (Lg, **B**) are shown as dots connected by the distance between the measurements. The dashed lines represent median values of the per-virus mean values of all CVV or WT viruses tested, while shaded areas (purple [CVV] or green [WT]) indicate standard error. D indicates the log differences between the measurements. Missing values indicate a mismatch in titration units between WT and CVV and were excluded from analysis with the exception of CDC-RG, RG2, and IBCDC-1 Tr, in which no specimen was collected for titration.

In contrast to NW and nasal turbinate specimens, a more pronounced attenuative effect was observed among CVV strains compared to WT viruses in tissues more distal from the nares (Fig. 4). For both trachea and lung specimens, median values for CVVs were at the limit of detection (Table S1). Similar to upper respiratory tract specimens, the degree of attenuation varied depending on the parental strain; however, CVVs overall had statistically significant lower day 3 p.i. viral titers compared to WT strains for both trachea (*P* = 6.3e-3) and lung (*P* = 2.3e-7) specimens (Table S1). In particular, the mean virus titers in lung tissues were >2.5 logs lower among CVV compared with WT strains, with the largest single mean WT-CVV pair reduction of viral titer in this specimen (>6 logs for IBCDC-1).

**Fig 5:**
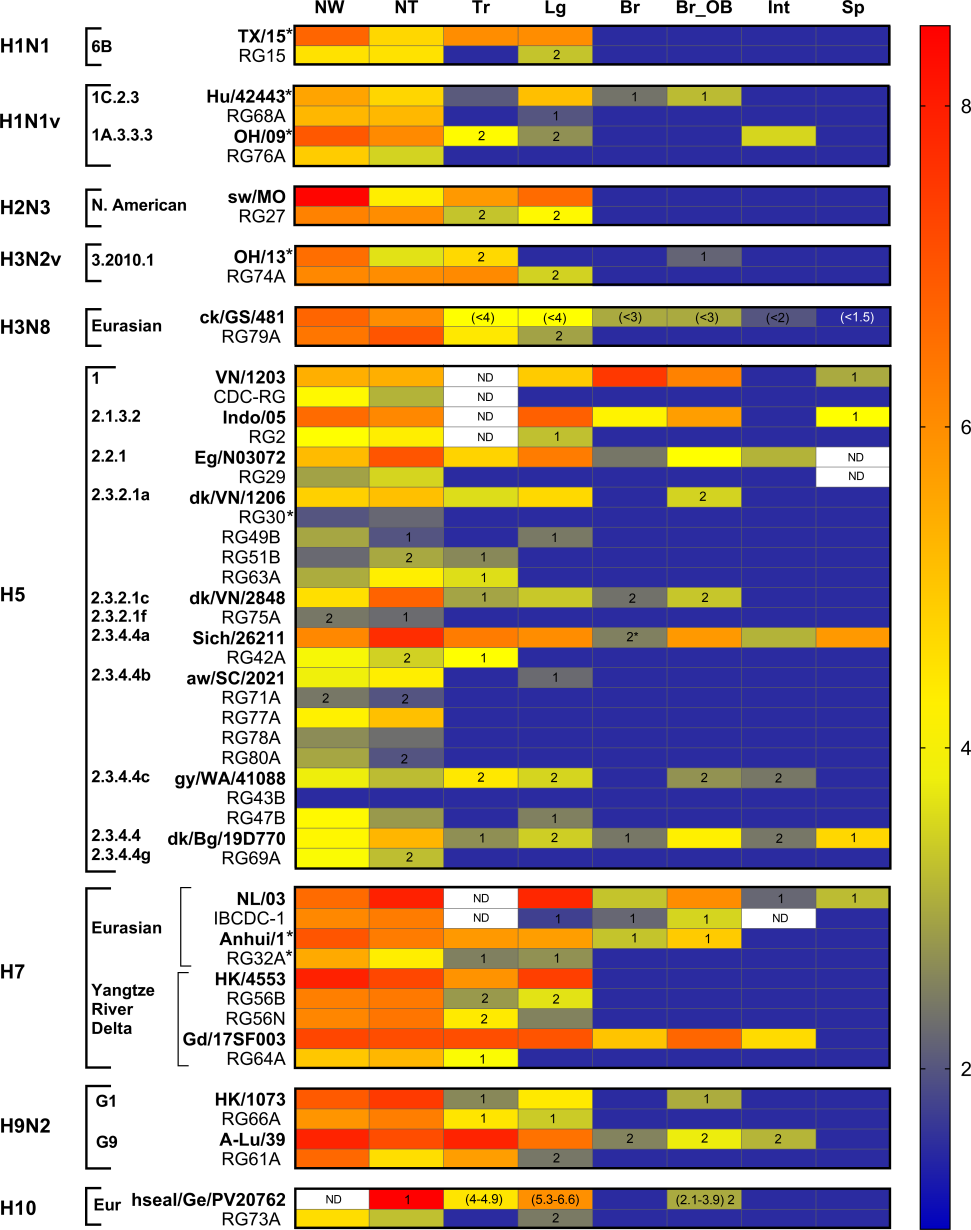
Heat map of virus titers found in ferrets inoculated with wild-type viruses (WT) or their respective CVVs. The colors and numbers in the gradient ruler represent the range of titers, from low (blue) to high (red). Titers are expressed EID_50_/mL or gram, PFU/mL or gram (*), or TCID_50_ (H10, H3N8), limit of detection: log_10_ 1.5 EID/mL or gram, 1.0 PFU/mL or gram, 1.0 TCID_50_/mL or gram. Numbers inside cells denote the number of ferrets out of 3 that had detectable virus in the specific tissue. No number in cells indicates that virus titers were detected in 3 out of 3 ferrets. ND, not determined. The numbers within parentheses show titer values (in TCID_50_) reported by outside investigators (references in Table 1). NW, nasal wash; NT, nasal turbinates; Tr, trachea; Lg, lungs; Br, brain; Br_OB, olfactory bulb; Int, intestines; Sp, spleen.

Extrapulmonary spread of IAV is not uncommon among HPAI viruses that cause severe disease. Accordingly, mean viral titers of CVVs were also significantly reduced in brain (*P* = 1.1e-2) and olfactory bulb (*P* = 3.1e-5) tissues (Table S1). Furthermore, frequency of virus detection was reduced in these and additional extrapulmonary tissues sampled, including the intestine and spleen (Fig. 5). Taken together, these results further support that CVV strains can maintain a capacity for replication throughout the mammalian respiratory tract though the frequency and magnitude of virus replication is reduced compared to parental WT strains. These results align with established guidelines, which indicate that the replication of the virus and the associated clinical symptoms should resemble those produced by the attenuated PR8 parent virus, while being less severe than disease caused by the corresponding WT zoonotic virus (7).

### Predictive magnitude of variables for IAV CVV attenuation

**Fig 6:**
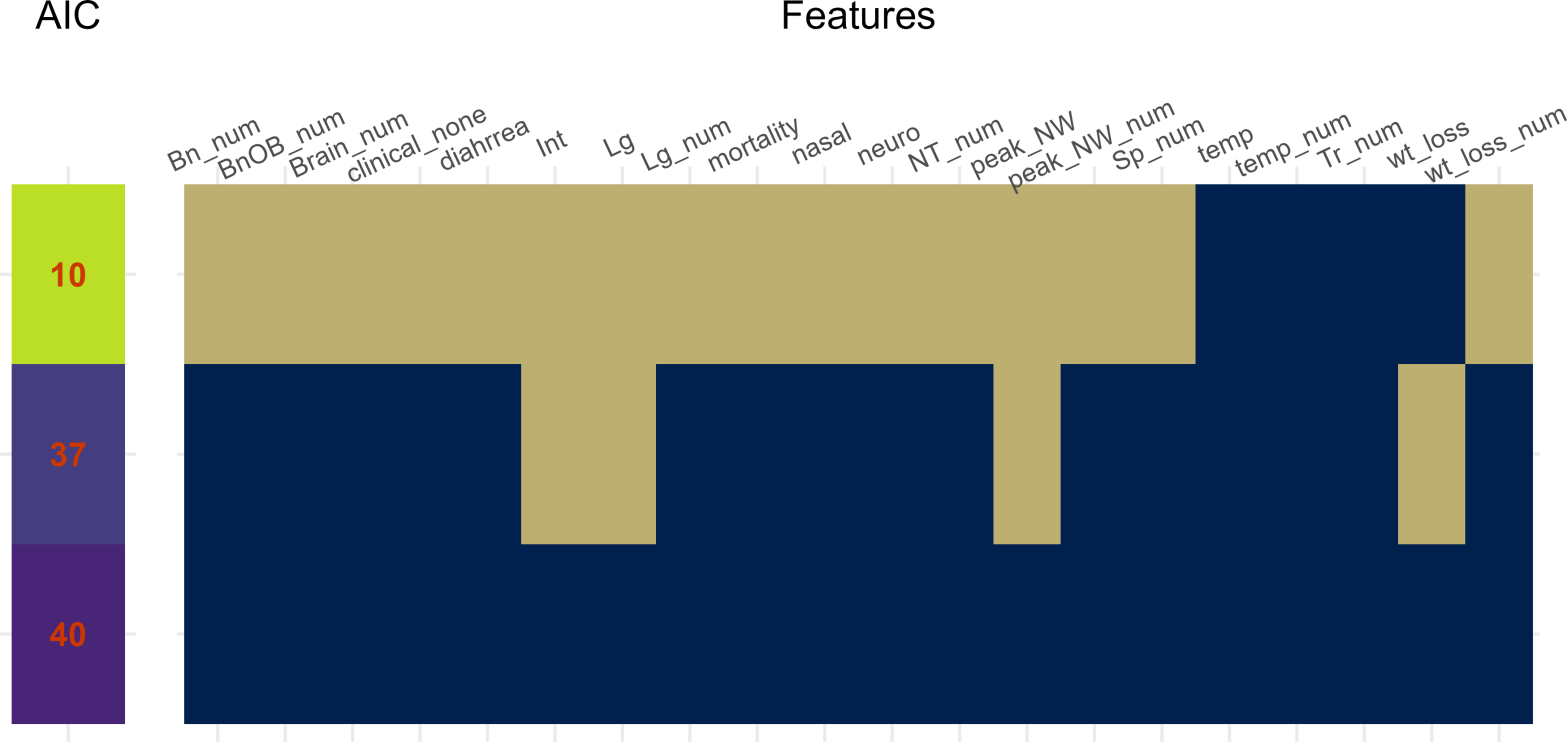
Strength of generalized linear models (GLM) with variable combinations of clinical and virological metrics of attenuation. Models with different combinations of features were ranked based on Akaike Information Criterion (AIC), where low AIC values indicate top performing GLM. Heat map depicts inclusion or exclusion of different variables in each model shown. Lower AIC values indicate higher strength (colored green) relative to higher AIC values (colored purple). Features for all models are shown as either dark blue (inclusion), or tan (exclusion). Individual definitions of features tested are provided in Table S3; for the highest-performing model, features were wt_loss (mean maximum weight loss), temp_num and weight_loss_num (number of ferrets with detectable temperature rise or mean maximum weight loss, respectively), and Tr_num (number of ferrets with virus detected in trachea). Full scope of all model metrics for models are reported in Table S5.

The evaluations above identified many independent variables that could potentially predict IAV CVV attenuation. We used generalized linear models (GLM) to assess different combinations of features to identify those elements most strongly associated with prediction of CVV attenuation. To do this, the evaluation of key variables for predictive CVV feature selection was performed using Ridge and Lasso regression models (Table S4 and S5), and the predictive strength of each model was determined by ranking each based on Akaike Information Criteria (AIC) where lower AIC values indicate higher-performing models. Models were tested with a full panel of 30 independent variables (full list in Table S3), spanning quantifiable virological titer and clinical measurements, and measures of the relative frequency of detection of these virological and clinical measurements (as indicated as _num, giving a number from 0 to 3 out of 3 inoculated ferrets in the group). Fig. 6 specifies which variables were included in each tested iteration of the GLM shown, with blue squares indicating the model included this variable, and tan squares indicating that the variable was not included. The top performing GLM accounts for 100% of the variation in the response variable (R-Squared) (Fig. 6; Table S5). This model incorporates a mix of quantifiable clinical [mean maximum weight loss (wt_loss), mean maximum rise in body temperature (temp)] and frequency detection metrics [number of animals with temperature change (temp_num), number of animals with virus detected in trachea (Tr_num)] as predictor variables. Isolating single variables reveals a notable impact of wt_loss (R-Squared = 0.51) and temp (0.44), and a comparatively lower influence of temp_num (0.22) and Tr_num (0.20). When we examine the model with only clinical signs (wt_loss and temp, 0.62), or by removing Tr_num only (0.72) or temp_num only (0.62), we see some reduction in explanatory power, but most variability is still explained by wt_loss and temp, further emphasizing their impact. The Bayesian GLM results underscore the significance of these findings, indicating a 100% probability of significance and a large effect (99.98%) for temp, followed by temp_num (99.98%, 99.98%), wt_loss (99.92%, 77.5%), and Tr_num (97.98%, 95.95%). Interestingly, while absence of clinical signs (clinical_none) was not part of the final models, a GLM employing only this parameter yielded reasonable explanatory power (0.37), showcasing the pivotal role of clinical signs in assessing CVV attenuation (Table S5) and supporting inclusion of these parameters when performing studies of this nature. Taken together, these results indicate that clinical metrics, specifically aggregates of frequency of detection of clinical signs and not the specific experimental values themselves, were consistently associated with the highest predictive utility for CVV attenuation among all variables captured during CVV safety testing.

## DISCUSSION

IAV CVV development represents a critical component of pandemic preparedness, that has garnered support domestically and internationally (6), by facilitating rapid generation of high-yield vaccine preparations for use when a novel IAV becomes capable of sustained transmission in the human population. According to international zoonotic CVV manufacturing recommendations (6), CVV attenuation must be demonstrated in the ferret model, as defined by comparison with WT viruses or within pathogenicity standards, for instance, replication should be within a predefined range and restricted to the respiratory tract with an absence of extrapulmonary spread (7, 15). The data presented in this analysis supports that CVVs are consistently attenuated relative to WT strains in the ferret model. However, understanding the relative degree of attenuation achieved across a panoply of clinical and virological parameters, and how this attenuation may vary depending on the heterogeneity of IAV subtypes is key to moving forward in establishing standard thresholds for virulence in CVV safety testing in the future and thereby eliminating the need for a paired parental strain for comparison. Data science approaches permit a robust way to achieve these outcomes. We employed statistical and logistic regression approaches to identify critical variables associated with virus attenuation consistently present across a diversity of CVVs, ultimately identifying the frequency of clinical parameters (notably reductions in peak temperature and peak weight loss) as valuable metrics of virus attenuation independent of WT strain heterogeneity.

Prior studies have reported CVV attenuation phenotypes in ferrets with both seasonal and novel IAV (13, 15). However, while these studies support that IAV can lead to varied degrees of attenuation of CVVs, none of them analyzed a data set of this size, inclusive of many IAV subtypes, lineages, and clades. Furthermore, as the diversity of studies captured here have high uniformity across laboratory facilities where the work was conducted, consistent protocols under which the experiments were performed, and sourcing of ferrets from a consistent vendor, the overall consistency across parameters which can vary between institutions (39) makes this data set unique in the field. The use of linear regression to identify specific *in vivo* correlates with phenotypic outcomes has been used in the past (40–42), but not previously in the context of CVV characterization.

This study collectively supports that CVVs exhibit attenuated clinical signs and virological titers compared with well-matched WT strains in the ferret model. The variation of attenuation observed is not surprising given the extensive heterogeneity of strains present in the data set; the few instances where attenuation of virological parameters was not detected is likely, in part, reflective of the inherent variances of the outbred animal model, minor variances in collection of specimens for titration, differences in the timing of collection, and other considerations. The restriction of viruses within a single HA subtype (H5, see Table S2) when analyzed by GLM resulted in increased statistical significance across many of the parameters assessed. Subtype-specific stratification was not possible for other subtypes in the data set due to limited sample sizes. While the low pathogenicity of CVVs in ferrets is, in part, attributed to the presence of PR8 virus backbone sequences, these viruses are nonetheless capable of replication in ferrets. The PR8 virus primarily replicates in the upper respiratory tract of ferrets with limited systemic dissemination (12, 38). This feature, and its ability to replicate to high titer in embryonated eggs, makes it a suitable backbone for CVVs. However, alternative virus backbones should not be ruled out.

Distilling *in vivo*-generated data to discrete values necessary to conduct statistical modeling can be a challenge (43). As such, analyses were conducted with both quantifiable measures (percent weight loss and temperature rise, both of which were normalized prior to analysis (37), or infectious virus titer) and frequency of detection assessments (number of ferrets that displayed the parameter out of the total per group). It is interesting that frequency measures, and not quantifiable values, were often the most strongly associated with statistically meaningful outcomes when assessing attenuation of CVVs relative to WT strains. Group sizes were necessarily small for these experiments though appropriate for the conclusions drawn (44); it is expected that these frequency assessments would still maintain high value if group sizes were increased. Future studies assessing the potential utility of summary metrics not included in this work, such as area under the curve, to capture overall viral shedding in NW specimens during the acute phase of infection, would be of interest.

The current study has limitations of note. The analyses conducted here were restricted to one institution; similar comparative aggregate work would be important to perform at other institutes conducting this work to ensure the metrics most associated with attenuated phenotypes in CVV are independent of experimental inoculation or laboratory-specific protocols. For clinical parameters, we only examined peak values and did not look at the frequency or duration of detection during the acute phase of infection, nor did we evaluate CVV transmission. There were selected instances (Table 1) where the WT virus used for comparison differed from the CVV (primarily by differences in lineages within the same clade, or different donor strains within the same lineage/clade); these decisions were made to adhere to the 3 R’s (reduction, refinement, replacement) governing animal research (45) so that additional ferrets were not used solely for attenuation comparison purposes when possible. Both cell-based (PFU) and egg-based (EID_50_) titrations were used to quantify infectious virus among wild-type and CVV viruses in this study due to strain- and subtype-specific replication capacities. Statistical analyses to assess relative degree of attenuation in viral titer between WT and CVV strains were only conducted for pairs for which the same titration matrix was used across the pair, to eschew any matrix-specific confounding (46). The majority of ferret inoculations were conducted with 10^6^ infectious units with few exceptions (footnotes in Table 1); however, all ferrets were inoculated with a consistently high dose of virus permitting data aggregation across this parameter (47). Although gross pathology scores are determined during pathogenicity tests for comparison to WT (7), we did not include them in our analyses due to interpretation variability, descriptive nature, and lack of standardization. Instead, we focused on objective metrics that could be quantified with laboratory tests that provide reliable and replicable data.

Our study supports the findings of Chen et al., which reviewed over 15 years of chicken pathogenicity tests on H5 and H7 CVVs, showing consistent attenuation in this species (48). Although USDA still requires select agent exclusion of H5 and H7 CVVs, chicken lethality testing as an exclusion method was removed by the Division of Agricultural Select Agents and Toxins (DASAT) in 2018, as long as trypsin dependency is demonstrated and genomic sequence confirms consistency with LPAI, thereby reducing the testing timeline for rapid vaccine responses. However, pathogenicity testing in ferrets is still a WHO recommendation for CVVs derived from both high and low pathogenicity viruses (7). The high increase in the number of zoonotic influenza viruses with pandemic potential over the past decade has led to a rise in CVV production and WT virus testing, drawing renewed attention toward the development of standards for ferret pathogenicity tests to streamline CVV evaluations so that vaccines can proceed in a timelier manner while drastically reducing the number of ferrets required for testing. Our analysis of cumulative data from pathogenicity tests conducted in ferrets has confirmed that these viruses are attenuated in these animals. This finding could potentially reduce the need for WT virus testing and enable more tailored biosafety assessments of CVVs in specific situations. 

*In vivo* experimentation represents a cornerstone of biomedical research, and it is not anticipated that the use of ferrets to support CVV development will be eliminated entirely. Considering the unpredictable nature of IAV, it is likely that future, emerging influenza strains chosen for CVV development will include viruses with alterations in receptor binding specificity or other changes related to the virus backbone or adaptation markers that might modify the degree of attenuation observed. Ferret safety testing will undoubtedly be necessary in these scenarios. By understanding which parameters are most substantially and consistently attenuated in CVVs generated under standard protocols, these analyses represent a critical step in developing attenuation standard thresholds that may be consistently applied to IAV CVV testing, thereby improving the timeliness and efficiency of this critical component of pandemic preparedness.

## MATERIALS AND METHODS

### WT and CVV development

WT IAV used for comparison analyses against their corresponding CVVs are listed in Table 1. Virus stocks were propagated in the allantoic cavities of 10–11 day old embryonated chicken eggs at 35–37°C for 24–48 h, minimizing the number of passages in order to avoid deleterious mutations (18, 24). Pooled allantoic fluid was clarified by centrifugation and aliquots were exclusivity tested by real-time reverse transcription (RT)-PCR to rule out the presence of other subtypes of influenza virus and stored at −80°C until use. Viral infectivity was determined by the EID_50_ end point inoculation into embryonated eggs or by plaque forming units (PFU) (49). The references for data obtained from outside laboratories are provided in Table 1.

The generation of reassortant viruses was performed according to guidance for development of vaccine reference viruses (7) and described in (15). All studies performed at CDC with live virus were conducted under biosafety level 3 containment, including enhancements required by the U.S. Department of Agriculture and the Federal Select Agent Program (50).

### Ferret pathotyping experiments

Ferret experiments were performed under the guidance of the Centers for Disease Control and Prevention’s Institutional Animal Care and Use Committee in an AAALAC International-accredited animal facility. Adult male Fitch ferrets, 6–12 months of age (Triple F Farms, Sayre, PA) and serologically negative for currently circulating influenza viruses were used in this study. Ferrets were intranasally (i.n.) inoculated with 10^6^ PFU or EID_50_ of virus diluted in PBS in a 1 mL volume unless otherwise specified in Table 1. The details for experiments using WT viruses can be found in references provided in Table 1. Studies using CVVs were performed according to established protocols and guidelines (15, 24, 51). For each CVV, three inoculated ferrets were monitored for 14 days for clinical signs of infection (including weight loss, fever, lethargy, sneezing, nasal discharge, and neurological dysfunction); any ferret that lost >25% of preinoculation body weight or exhibited signs of neurological involvement was humanely euthanized. Body temperature was measured once daily, most often in the mornings, using subcutaneous implanted transponders (BMDS). NW specimens were collected every-other-day for 7 days for determination of virus titer. Three additional CVV-inoculated ferrets were euthanized on day 3 p.i. for tissue collection and determination of virus spread (7). All viral titers are reported as log_10_ infectious units/mL or g. The titration limit of detection was 10^1.5^ EID_50_ or 10 PFU.

### Data aggregation for comparative analyses

Aggregated data employed a minimum of *n* = 3 ferrets per virus for all parameters. Temperature reports the mean peak rise over preinoculation baseline temperature in degrees C between days 1–9 p.i. Weight loss reports the mean peak weight loss as a percentage from preinoculation baseline between days 1 and 9 p.i. NW titer reports the mean maximum titer between days 1 and 7 p.i. from each ferret such that the reported average may be inclusive of multiple collection days. For all tissues, titer averages reported in figures include only values that were above the limit of detection; the total sample size for each WT-CVV pair is specified in Table S1. Brain tissue represents a pooled sample of anterior and posterior sections (when these were titered separately in source experiments, these titers were averaged for inclusion in this study). Lung titers are inclusive of pieces collected from all lobes. Tissues with viral titers and clinical measures (temp and weight loss) were designated a column (‘_num’) with the number of ferrets (out of 3 per group) that had a measurable titer for a frequency of detection value.

### Predictive CVV feature selection

The evaluation of key variables for predictive feature selection of CVVs employed Ridge and Lasso regression models through the R package glmnet v4.1.6 (52). Model performance was assessed using R-Squared values. With variables identified in regularization models, logistic regression generalized linear models were explored, incorporating the stepwise algorithm to pinpoint pivotal variables further for CVV prediction, utilizing AIC for model evaluation. The most effective GLM underwent Bayesian analysis using 4 MCMC chains and 2,000 iterations (with 1,000 burn-in) with the rstanarm v2.32.1 package (53). Statistical interpretation was facilitated using the report v0.5.8 package (54). Visualizations were generated using the packages tidyverse v2.0.0 (55), ggplot2 v3.5.0 (56), ggpubr v0.5.0 (57), gt v0.10.0 (58), and viridis v0.6.2 (59).

## Data Availability

Sequence data for the viruses reported here have been deposited in GenBank under the accession numbers listed in Table 2.

## SUPPLEMENTAL MATERIAL

Supplemental tables jvi.01023-25-s0001.xlsx Tables S1 to S5.

ASM does not own the copyrights to Supplemental Material that may be linked to, or accessed through, an article. The authors have granted ASM a non-exclusive, world-wide license to publish the Supplemental Material files. Please contact the corresponding author directly for reuse.
